# Effects of fermented soybean meal with *Bacillus velezensis*, *Lactobacillus* spp. or their combination on broiler performance, gut antioxidant activity and microflora

**DOI:** 10.5713/ab.21.0529

**Published:** 2022-04-29

**Authors:** C. F. Tsai, L. J. Lin, C. H. Wang, C. S. Tsai, S. C. Chang, T. T. Lee

**Affiliations:** 1 Department of Animal Science, National Chung Hsing University, Taichung 402, Taiwan; 2 School of Chinese Medicine, College of Chinese Medicine, China Medical University, Taichung, 404, Taiwan; 3 Central Union Oil Corporation, Taichung 436, Taiwan; 4 Kaohsiung Animal Propagation Station, Livestock Research Institute, Council of Agriculture, Kaohsiung 912, Taiwan; 5 The iEGG and Animal Biotechnology Center, National Chung Hsing University, Taichung, 402, Taiwan

**Keywords:** Antioxidant, Broiler, Fermented Soybean Meal, Performance, TCA-soluble Peptides

## Abstract

**Objective:**

A series of experiment were conducted to evaluate the effects of replacing a part of soybean meal (SBM) at 6% of broiler diets with fermented soybean meal (FSBM) obtained by single or two-stage fermentation by measuring growth performance, antioxidant activity in the jejunum and distal intestinal microflora.

**Methods:**

Soybean meal samples were prepared by single-stage fermentation using *Bacillus velezensis* (Bv) (FSBM_B_), or *Lactobacillus* spp. (as commercial control) (FSBM_L_). Additional SBM sample was prepared by two-stage fermentation using Bv and subsequently using *Lactobacillus brevis* ATCC 367 (Lb) (FSBM_B+L_). Enzyme activity, chemical composition, trichloroethanoic acid-nitrogen solubility index (TCA-NSI) and antioxidant activity were measured. Then, in an *in vivo* study, 320 Ross308 broilers were divided into four groups with *ad libitum* supply of feed and water. Four groups were fed either a corn-soybean meal diet (SBM), or one of fermented SBM diets (FSBM_B+L_, FSBM_B_, and FSBM_L_). Growth, serum characteristics, microflora, and the mRNA expression of selected genes were measured.

**Results:**

Compared to SBM, FSBM_B+L_ contained lower galacto-oligosaccharide, allergic protein, and trypsin inhibitor, and higher TCA-NSI by about three times (p<0.05). Reducing power and 1,1-diphenyl-2-picrylhydrazyl free radical scavenging ability correlated positively with the TCA-NSI content in FSBM. Growth performances were not significantly different among four groups. In jejunum of 35-day-old broilers, partial replacement of SBM by FSBM_B+L_ increased the activity of superoxide dismutase and catalase (*CAT*), and the FSBM_B_ group had the highest catalase activity (p<0.05). Partial replacement of SBM by FSBM increased relative mRNA expressions of nuclear factor erythroid 2-related factor 2 (*Nrf2*), heme oxygenase-1 (*HO-1*), and peptide transporter 1 (*PepT1*) (p<0.05); however, FSBM_B+L_ increased *CAT* mRNA level to 5 times of the control (p<0.05).

**Conclusion:**

Using Bv- and Lb-processed SBM through two-stage fermentation to partially replace 6% of diets will improve the gut's antioxidant activity under commercial breeding in broilers.

## INTRODUCTION

The by-product from soybean oil production, soybean meal (SBM) is the most common feed protein resource in the diet of farm animals. However, some well-known anti-nutritional factors (ANFs) in soybean meal, like soy structural protein, soy trypsin inhibitor (TI), soy agglutinin, non-starch polysaccharide, and soy galacto-oligosaccharide (GOS), will limit the nutrient utilization by animals [1] and induce gut inflammation, intensifying the damage of intestinal epithelial, thereby increasing the suffering of the farm animals from diseases and stress. In the poultry industry, especially broilers, soy protein is safely fed by adjusting the diet formula for fast body growth. To alleviate this situation, solid-state fermentation (SSF) becomes the most beneficial way to enhance SBM nutrient quality.

In the early years, the product development of fermented soybean meal (FSBM) focused on increasing *in vitro* protein digestibility, enhancing the utilization efficiency by the farm animal. However, recent years of research have shown that SSF decreases soy ANFs and increases the bioactive ingredients. Soy peptides, the hydrolysis products of soy structural protein contain 2 to 20 amino acid residues or more than 21 amino acids within the 3-dimensional structure [2] are the main bioactivity ingredients. For retaining the maximum nutrients and biological activity during the production, the best approach is hydrolysis by endo-protease or exo-proteases.

Soy peptide production can occur in two ways: enzyme addition like bromelain, papain, alcalase, flavourzyme, neutrase, transglutaminase, and through SSF by bacteria or fungi [3]. *Aspergillus* spp. are known for robust structural degradation by enzyme activity but delay the synthesis of the fermented products. *Bacillus* spp. are reported to provide more bioactive soy peptides with shorter SSF and improve the antioxidant activity. On the other hand, lactic acid bacteria (LAB) have difficulty to reducing the ANFs and increasing the bioactive ingredients through one-stage SSF fermentation [4]. Co-fermentation of FSBM by *Bacillus* spp., combined with other functional strains like LAB is a new trend used to improve the protein source in livestock diets.

These days, the aim of the development of FSBM for livestock is to enhance the small soy peptide (<10 kDa) content and to appropriately replace SBM in the diet. Soy peptides of molecular weights (MW) between 5 to 10 kDa showed better inhibition of pro-inflammatory protein markers like interleukin-6, nuclear factor κB, and decreased the production of oxides, while soy peptides of MW <3 kDa may be highly correlated to the 1,1-diphenyl-2-picrylhydrazyl (DPPH) radical scavenging abilities, which could help the tissue reduce the oxide stress and restore normal antioxidant function [5]. A recent report about the partial replacement of FSBM in poultry diets showed that 3% or 6% [6] could improve the growth performance, regulate the intestinal flora, increased villus height, and reduce the oxide stress while improving the antioxidant activity at target tissues like the colon, jejunum, ileum, liver, or breast. So far, there are only a few reports on FSBM-derived bioactive soy peptides with antioxidant property, especially the two-stage FSBM, on the poultry’s performance, gut oxide stress, and microbiology. A recent study showed that *Bacillus stearothermophilus* FSBM containing about 60 g/kg of soy peptides partial replacement diet by 5%, 10%, to 15% improved the intestinal morphology and microflora of the broilers, although it did not clarify the mode of regulation of FSBM and peptides on the antioxidant capacity of animals [7]. Continuing recent research, besides growth performance and intestinal flora, the current study focused on associating soy bioactive peptides derived from FSBM with gut oxide stress in broilers.

## MATERIALS AND METHODS

### FSBM preparation

Two strains were enriched in 1×10^9^ CFU/mL: *Lactobacillus brevis* ATCC 367 (Lb) isolated from kimchi, and cultured in de Man, Rogosa and Sharpe (MRS) broth at 30°C, in anaerobic conditions for 48 h; *Bacillus velezensis* (Bv) was screened by our lab and cultured in Luria-Bertani (LB) broth at 37°C, aerobic for 24 h. Then, 50 g SBM available commercially from Central Union Oil Corporation (Taichung, Taiwan) was sterilized at 121°C for 15 min. After SBM cooled down, sterile distilled water was used to adjust initial moisture to 50% and fermented in two different conditions: one-stage FSBM fermented by inoculating 2.5% Bv in aerobic fermentation for 60 h (FSBM_B_), and two-stage fermentation by inoculating 2% Bv in aerobic fermentation for 24 h, and then inoculating Lb in for further anaerobic fermentation for 36 h (FSBM_L+B_). At the end of SSF, 1 g fresh fermented product was collected for determination of pH, cell plate count, and enzyme activities. The remaining products were dried at 55°C for 12 h, ground, and stored at −20°C for other analyses.

### Enzyme activity assay

One gram of fresh fermented product was mixed with 50 mM sodium acetate buffer on ice for 30 min, and the supernatant was used for the following analysis. Protease determination was modified from Toe’s method [8], and one unit was defined as hydrolysis of 1 mg/mL azocasein and change in 0.001 absorbance value. α-galactosidase determination protocol was modified according to Hati et al [9], and one unit was defined as hydrolysis of 2 mM p-Nitrophenol α-D-Galactopyranoside and release 1 μM p-nitrophenol. Following assays were conducted according to Miller [10]: One unit of cellulase was defined as consumption of 5 mg/mL CMC to generate 1 μM of reducing sugar; one unit of β-glucanase was defined as consumption of 4 mg/mL β-glucan to generate 1 μM of reducing sugar; one unit of β-mannanase was defined as consumption of 3 mg/mL locust bean gum to generate 1 μM of D-mannose; one unit of xylanase was defined as consumption of 10 mg/mL beechwood xylan to generate 1 μM of D-xylose.

### Composition analysis of fermented soybean meal

The FSBM was dried at 105°C for 4 h. The crude compositions included dry matter (DM), crude protein, ether extrac, ash, total phosphorus, and calcium, and their levels were measured according to AOAC [11]. The quantitation was carried out using commercial enzyme-linked immunosorbent assay (ELISA) kits for allergen protein (Biofront, Tallahassee, FL, USA) and trypsin inhibitor (Eurofins Immunolab, Kassel, Germany). Trichloroethanoic acid-nitrogen solubility index (TCA-NSI) was measured according to Xie’s method [12]. Supernatant extracted from FSBM was dissolved in 10% TCA and the nitrogen content was measured by the Kjeldahl method [11]. At the end of the test, TCA-NSI was calculated with the following equation:


$$\text{TCA-NSI }(\%)=({\text{N}}_{1}/{\text{N}}_{0})\times 100$$

N_1_ is nitrogen content from FSBM extracted with 10% TCA (mg), and N_0_ is nitrogen content from FSBM (mg).

### High-performance liquid chromatography assay

One gram of sample was extracted with 10 mL deionized water, homogenized at 50°C in a hot bath for 2 h. To the supernatant, 9% Barium hydroxide and 10% Zinc sulfate (400 μL) were added, mixed well, and centrifuged at 13,000 rpm for 5 min. The supernatant was freeze-dried, restored using 50% acetonitrile, and filtrated through a 0.22 μm filter before use. The levels of galacto-oligosaccharides (stachyose, raffinose) were measured according to Yin’s method [13] with high-performance liquid chromatography (HPLC) (Hitachi, Kyoto, Japan) equipped with a pump (CM 5110), and an auto-sampler (CM 5210). A column (ZORBAX carbohydrate, 4.6 mm×150 mm, 5 μm) was used; it was connected with a column oven (CM 5310; 30°C), and a refractive index (RI) detector (CM 5450; 35°C). The mobile phase was 70% acetonitrile at a flow rate of 1 mL/min for 40 min. The stachyose tetrahydrate, D (+)-raffinose pentahydrate, D (+)-sucrose, D (+)-galactose, and D (+)-fructose were used as standard at 6, 3, 1.5, 0.75, 0.375, 0.1875 mg/mL, respectively.

### Assay for antioxidant activity

The sample was extracted following Handa et al [14]. Briefly, samples were extracted in 10 volumes of 50% ethanol at room temperature (25°C). The supernatant was diluted to 100 mg/mL^−1^. Butylated hydroxytoluene (BHT) was used as the assay standard for reducing power and free radical scavenging ability, and ethylenediaminetetraacetic acid disodium salt dihydrate (EDTA-2Na) for ferrous ion chelating ability of about 100 to 1 mg/mL. The reducing power assay was conducted as described by Oyaizu [15]. One milliliter of diluted sample was dissolved in phosphate buffer (PBS) and 1% potassium ferricyanide (2.5 mL), mixed well, and kept at 50°C in a hot bath for 20 min. At the end of the reaction, 10% TCA solution (2.5 mL) was added, homogenized, and 2.5 mL of this supernatant was mixed with deionized water (2.5 mL) and 0.1% ferric chloride (0.5 mL). The absorption was then measured at 700 nm. The method for determining DPPH free radical scavenging assay was as described by Gyamfi et al [16]. One volume of 1 mM DPPH solution was mixed with four volumes of the diluted sample, and allowed to stand at room temperature for 30 min. The absorption of the supernatant was measured at 517 nm and the inhibition was calculated as follows:


$$\text{Inhibition }(\%)=\left[1-\frac{\text{Sample absorp at }517\text{nm}}{\text{BHT absorp at }517\text{nm}}\right]\times 100\%$$

The ferrous ion chelating assay was determined using the method described by Yuris et al [17]. One milliliter of diluted sample was dissolved in 50% ethanol (9.25 mL) and 2 mM Ferrous chloride tetrahydrate (25 μL), mixed well and allowed to stand at room temperature for 30 s. Next, 5 mM ferrozine (50 mL) was added and allowed to react at room temperature for 10 min. The absorption of the supernatant was measured at 562 nm and the chelating abilities were calculated as follows:


$$\text{Chelating ability }(\%)=\left[1-\frac{\text{Sample absorp at }562\;\text{nm}}{\text{EDTA}-2\text{Na absorp at }562\;\text{nm}}\right]\times 100\%$$

### Animal experimental design and housing

The feeding trial was conducted during summer with an average environmental temperature of 30°C±2°C and average environmental humidity of 77%±11% at the Experimental Husbandry Farm (National Chung Hsing University, Taiwan), and all protocols in accordance with the guidelines by the Animal Care and Use Committee in NCHU (IACUC: 109-055). Three-hundred and twenty 1-day-old Ross 308 broilers with initial weight 44±1.2 g were categorized into four treatment groups with four replicates of corn-SBM feeding group (SBM), diets replacement by 6% of FSBM_B_, FSBM_B+L_, and an FSBM fermented by *Lactobacillus* spp. (as commercial control) (FSBM_L_). All chickens were raised in a temperature-controlled house, per pen (2×4 square meters) paved with rice bran litter. The feeding formula in Table 3 (A and B) was configured according to NRC [18] for starter (day 1 to 21), and finisher (day 22 to 35) with equal amounts of protein and energy. The feed and water were provided *ad libitum*. Per pen, the temperature was 34°C±1°C on day 1 and slowly reduced to 27°C±1°C at day 7 and maintained at this temperature until the end of the trial.

### Growth performance and sample collection

The body weight (BW) and feed intake (FI) for each group with replicate were measured on days 21 and 35. The body weight gain (BWG), feed conversion rate (FCR), and performance efficiency factors [19] were calculated later (the formula is described below Table 4). The survival rate was recorded daily. On day 35, six birds within average weight from each group were selected for euthanizing and sampling. To collect microbes from ileum and cecum, these birds were not fasted. Following electricity slaughter the intestines were removed and cleaved to obtain digesta and stored in PBS. The digesta of 0.1 g were homogenized and diluted in PBS. The MRS agar and CHROMagar ECC (CHROMagar, Paris, France) were used, respectively, for the measurement of lactic acid bacteria and coliform bacteria. Plates were cultured in anaerobic conditions at 37°C for 48 h and living cells were counted. For other analyses, birds were kept on fast for 24 h, euthanized by cervical dislocation, and the middle section of jejunum (2 cm) were collected. After rinsing by PBS, the sections were frozen in liquid nitrogen, stored at −80°C until used for antioxidant capacity assay and RNA extraction.

### Serum biochemistry characteristics

On day 35, eight birds from each group were collected, and serum sample from the wing vein was intravitally extracted. The serum sample was kept at 4°C for 4 h and centrifuged at 3,000 rpm at 4°C for 10 min. Then, took 1 mL from each serum sample for an antioxidant capacity assay. The rest of the serum samples were measured using the automatic biochemical analyzer (7150 auto-analyzer; Hitachi, Tokyo, Japan) as follows: The glucose content (GLU), blood urea nitrogen (BUN), urea (UA), serum glutamic-oxaloacetic transaminase, serum glutamic-pyruvic transaminase, total protein, albumin, globulin, alkaline phosphatase, cholesterol, high-density lipoprotein-cholesterol, and low-density lipoprotein cholesterol.

### Antioxidant capacity in serum and jejunum

The antioxidant enzyme activity assay for serum and jejunum sample were measured by commercial ELISA kits (Cayman, MI, USA) for glutathione peroxidase (GSH-Px), superoxidase (SOD), and CAT.

### Quantitative real-time reverse transcription-polymerase chain reaction analysis

The sample of 0.1 g stored in the RNA shield (Zymo research CO., Tallahassee, CA, USA), was macerated in a lysis tube (Zymo research CO., USA) with RNAzol (Molecular Research Center, Cincinnati, OH, USA). The supernatant was later extracted using the commercial kit’s protocol (Zymo research CO., USA). RNA was reverse transcribed using a Prime Script RT reagent Kit with gDNA Eraser (Applied Biosystems, Beverly, MA, USA). The real-time reverse transcription-polymerase chain reaction (RT-PCR) analysis was carried out on the StepOnePlus Real-Time PCR System (Thermo Fisher, Waltham, MA, USA). The dilution of cDNA and primer were according to protocol included in the instrument. In short, 2× SYBR GREEN PCR Master Mix-ROX (Applied Biosystems, USA), cDNA, deionized water, and each primer were mixed in a ratio of 5:1.2:1.8:1. To assess RT-PCR performance, the relative mRNA expression level was measured by the 2^−ΔΔCt^ method. While *β-actin* was used as the housekeeping gene, gene-specific primers were according to the genes of *Gallus gallus* (chicken) as follows: *Beta-actin* (*β-actin*, 5’-CTGGCACCTAGCACAATGAA-3’ and 5’-ACATCTGCTGGAAGGTGGAC-3’); peptide transporter 1 (*PepT1*, 5’-CAGGGATCGAGATGGACACT-3’ and 5’-CACTTGCAAAAGAGCA GCAG-3’); nuclear factor erythroid 2 like 2 (*Nrf2*, 5’-GGAAGAAGGTGCGTTT CGGAGC-3’ and 5’-GGGCAAGGCA GATCTCTTCCAA-3’); heme oxygenase 1 (*HO-1*, 5’-AGCTTCGCACAAGGAG TGTT-3’ and 5’-GGAGAGGTGGTC AGCATGTC-3’); catalase (*CAT*, 5’-CCACGTGGACCTCTTCTTGT-3’ and 5’-AAACACTTTCGCCTTGCAGT-3’); glyceraldehyde 3-phosphate dehydrogenase (*GAPDH*, 5’-CCTCTCTGGC AAAGTCCAAG-3’ and 5’-CATCTGCCCATTTGATGTTG-3’).

### Statistical analysis

All data were analyzed by using SAS software (SAS 9.4, 2016; SAS Institute Inc., Cary, NC, USA) with analysis of variance. Differences between treatment means were separated using Duncan’s multiple range test with p-value<0.05. The experiment for FSBM analysis was conducted in triplets with three independent fermented products.

## RESULTS

### Composition and enzyme activity of one or two-stage-FSBM products

The analyses of enzyme activities are shown in Table 1. Activities of protease and α-galactosidase were not detected in FSBM_L_. The highest protease activity of 4.79 U/g was detected in FSBM_B_. FSBM_B+L_ has significantly higher cellulase activity (2.88 U/g) compared to FSBM_L_ (2.27 U/g) and FSBM_B_ (2.51 U/g) (p<0.05). The composition of SBM, FSBM_L_, FSBM_B_, and FSBM_B+L_ are shown in Table 2. All three treatments significantly reduced GOS and allergen protein SBM (p<0.05). Stachyose content was not detected in FSBM_B_ and FSBM_B+L_. FSBM_B_ has the lowest content in allergen protein (183 mg/g DM) and TI (0.43 mg/g DM). Following Tables 1 and 2, Bv was found to hydrolyze proteins and rising level of TCA-NSI by 3-times compared to that in SBM (p<0.05).

### Antioxidant activity of one or two-stage-FSBM *in vitro*

**Figure 1:**
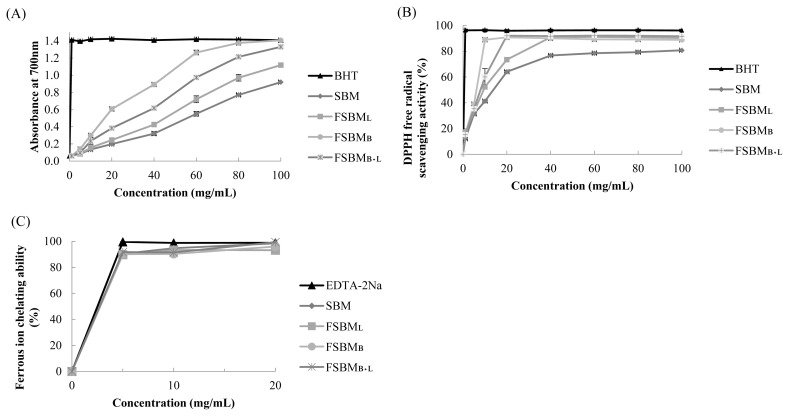
*In vitro* antioxidant assay for reducing power (A), DPPH free radical scavenging activity (B), and ferrous ion chelating ability (C) of FSBM at different concentration. DPPH, 1,1-diphenyl-2-picrylhydrazyl; FSBM, fermented soybean meal; SBM, soybean meal; FSBM_L_, commercial control; FSBM_B_, SBM one-stage fermented by Bv; FSBM_B+L_, SBM two-stage fermented by Bv and Lb. Each value represents the mean±standard deviation (n = 3).

Figure 1a shows the results of the estimation of reducing power. The absorbance at 700 nm by 100 mg/mL of FSBM_L_, FSBM_B+L_, and FSBM_B_ was 1.211, 1.393, and 1.407, respectively. Figure 1b presents the results of DPPH free radical scavenging activity. The highest scavenging activity was 92.1% at 20 mg/mL by FSBM_B+L_. Figure 1c shows the results of ferrous ion chelating ability. FSBM_L_, FSBM_B_, SBM, and FSBM_B+L_ reached the highest chelating ability at about 89.8%, 90.2%, 90.5%, and 91.7% at the concentration of 5 mg/mL, and there was no change when the concentration was further increased.

### Growth performance and blood biochemistry

The results of growth performance are presented in Table 4. Overall, the BW, BWG, FI, and FCR were not significantly different among the three treatments and control groups (p> 0.05). For blood biochemistry, the serum GLU in FSBM_L_, FSBM_B_, and FSBM_B+L_ were increased to 251 mg/dL, 242 mg/dL, 237 mg/dL, and 207 mg/dL, respectively, but not significantly different among the three treatments (p>0.05). The results of other blood biochemical parameters are presented in Table 5.

### Intestinal microflora concentration

Table 6 shows the results of the LAB and the coliform counts. The LAB counts in the cecum, FSBM_B_ (8.69 Log CFU/g) and FSBM_B+L_ (8.80 Log CFU/g) were significantly higher than in FSBM_L_ (7.85 Log CFU/g) (p<0.05), but there was no significant difference among the three treatments and control groups in the ileum (p>0.05). The number of coliforms in only FSBM_B+L_ was significantly lower than in FSBM_L_ and FSBM_B_ (p<0.05). In the cecum, compared with the L/C ratio of SBM, that of FSBM_B_ and FSBM_B+L_ were significantly higher 7% and 17%, and FSBM_B+L_ had the highest ratio.

### *In vivo* antioxidant activity

According to Table 7, compared to SBM, all the treatments showed an increase in malondialdehyde concentration (p> 0.05) in the jejunum and serum, but with increased antioxidant activity. In the jejunum, FSBM_B+L_ had significantly higher SOD activity than that in SBM (p<0.05), and other treatments showed a comparable trend to that of SBM (p<0.05); the CAT activity was significantly different among the groups (p<0.05), but FSBM_B_ was higher than in the others. In the serum, both FSBM_B_ and FSBM_B+L_ had significantly increased CAT activity compared to that in SBM and FSBM_L_ (p<0.05), but there was no significant difference between FSBM_B_ and FSBM_B+L_ (p>0.05).

### Relative mRNA expression in the jejunum

**Figure 2:**
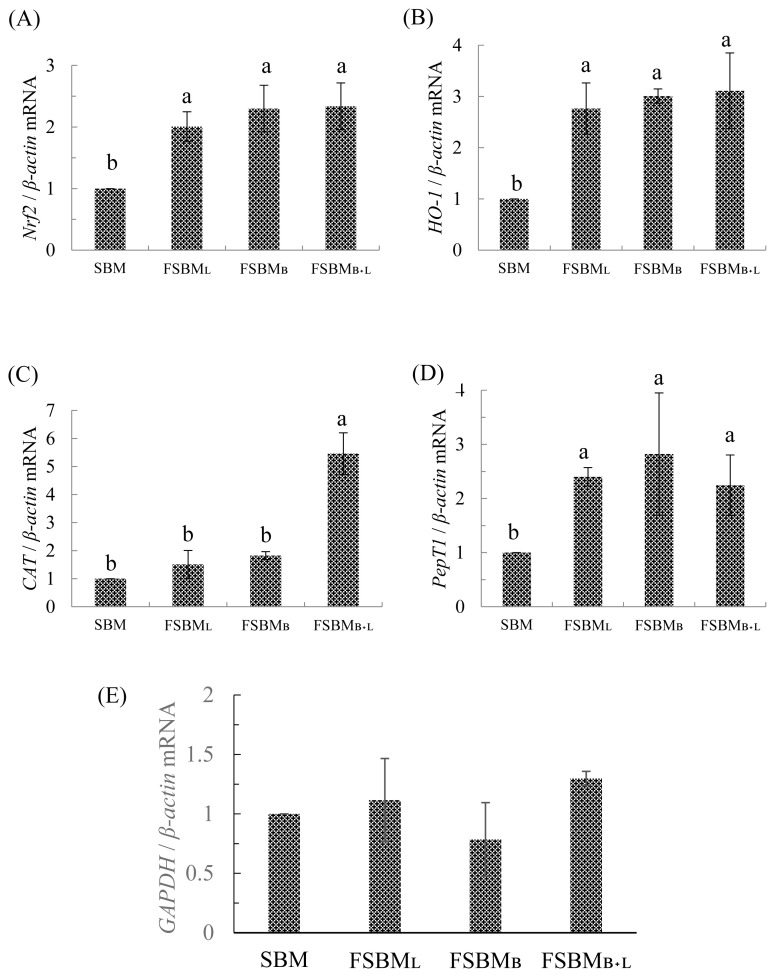
Effect of partial replacement of diet with FSBM on jejunum’ s relative mRNA expression of genes related to (A) *Nrf2*, (B) *HO-1*, (C) *CAT*, (D) *PepT1*, and (E) *GAPDH* of 35d-old broilers. FSBM, fermented soybean meal; *Nrf2*, nuclear factor erythroid 2-related factor 2; *HO-1*, heme oxygenase-1; *CAT*, catalase; *PepT1*, peptide transporter 1; *GAPDH*, glyceraldehyde 3-phosphate dehydrogenase; SBM, soybean meal; FSBM_L_, commercial control; FSBM_B_, SBM one-stage fermented by Bv; FSBM_B+L_, SBM two-stage fermented by Bv and Lb. Each value represents the mean±standard deviation (n = 5). ^a,b^ Means with different letters differed significantly (p<0.05).

The relative mRNA expression is presented in Figure 2 A to E. Compared to SBM, all three treatments had significantly upregulated *Nrf2* and *HO-1* by 2- and 3-times, respectively (p<0.05). *CAT* in the FSBM_B+L_ was significantly upregulated 5 times than in other groups (p<0.05). On the other hand, the *PepT1* in all three treatments was 2 times significantly upregulated than in SBM (p<0.05) but was not significantly different among FSBM_L_, FSBM_B_, and FSBM_B+L_ (p>0.05). All of treatments have no significant difference at *GAPDH* related expression (p>0.05).

## DISCUSSION

To the best of our knowledge, this is the first report of the use of *Bacillus velezensis* (Bv) in combination with *Lactobacillus brevis* for SBM fermentation by selected strains. The change in SBM composition was due to Bv fermentation. Also, Bv could decrease the antigen activity of structure protein and TI, an effect similar to that reported by Liu [20]. We discovered a greater decrease in NDF content in FSBM_B_ compared to that in FSBM_L_, suggesting that Bv may release more simple sugars by hydrolyzing hemicellulose and pectin. Furthermore, the preliminary judgment of the content of soy peptides was according to TCA-NSI and was more than 10% [21]. In our experiment, the DPPH radical scavenging ability was positive in correlation with TCA-NSI content but that did not include the ferrous chelating ability, which is sufficient to demonstrate its representativeness [22].

The advantage of FSBM for meat farm animals is that it has reduced anti-nutrition factors and improved protein digestibility, which then increases the economic value due to improved BWG and FCR [23]. In our study, FSBM_B+L_ showed the highest survival rate and lower FCR of the broilers than in other treatment groups and SBM control, but not the most obvious improvement compared to other studies.

In birds, the BUN and UA content were in response to the nitrogen metabolism [24]. In our study, the BUN and UA in treatment groups did not increase significantly, although a few earlier studies showed the decrease in UA content in the serum of broilers after feeding a 6% replacement FSBM diet [6]. Wu [7] considered that serum glutamic-oxalocetic transaminase (GOT) is associated with the body’s antioxidant ability. Accordingly, in our study, the FSBM_B_ with the highest TCA-NSI content and antioxidant ability had slightly reduced GOT whereas FSBM_B+L_ did not cause any negative effect to the broilers’ body, while FSBM_L_ exhibited increased GOT activity.

The gut microflora mainly colonizes in the distal intestine of poultry, including the ileum, cecum, and colon. GOS from soybean is harmful to the complex microflora, especially potential pathogens that develop in the distal intestine for monogastric animals because of the lack of α-galactosidase [9]. In addition, the status of protein digestion and absorption in the diet also affects the distribution of intestinal flora [25]. FSBM fermented by *Bacillus* spp., *Lactobacillus* spp., and *Saccharomyces* spp., combined with prebiotics could significantly affect the LAB content, which leads to lowered pH and coliforms, and increased villus height in the distal intestine [26]. FSBM contains hydrolysate including peptides, organic acids, and simple sugars that support native LAB growth and compete with pathogens. Also, FSBM feed with more soy peptide contents is helpful in regulating the microflora, especially the *Lactobacillus* spp. at the distal intestine, and reduced the *E. coli* in the cecum [7]. The FSBM**_L_** only reduce the pH value and increase the lactic acid bacteria count compared with SBM. Insufficient degradation of GOS and soybean protein may lead to the development of lactic acid bacteria in the distal intestine that is not as good as FSBM_B_ and FSBM_B+L_. This part is worth discussing in further study.

The FSBM treatment increased MDA concentration slightly but did not inhibit the activity of the antioxidant enzymes. On the other hand, the better slaughter rate by FSBM_B_ and FSBM_B+L_ (84.8%) than that of the control (83.4%) (data not shown), FSBM may reduce carcass lipid accumulation but needed further research support. Similar results were also observed by Yang et al [5] who reported increased CAT, SOD, and GSH-Px activities in mouse serum and liver. In the jejunum, all three FSBMs led to an increase in CAT activity, but SOD activity increased only in the FSBM_B+L_ group. Guo et al [27] points out that two-stage FSBM derived soy peptide with 2.5% partial replacement in the feed of broilers could regulate the SOD activity at the breast, and 7.5% partial replacement could decrease MDA and hydrogen peroxide concentration. We consider that the regulation of antioxidant activity in the jejunum was affected by the bioactivity of soy peptides, which were modified by fermented strains. Still, we must notice some factors from the fermented products such as protein level, protein degree of degradation [25], and other negative factors from processing like Maillard reactions [5], which could avoid feed and bacterial disorders leading to increased oxidative stress.

In Figure 2 (E), we use *GAPDH* related mRNA expression to fill a vacancy of feed composition analysis. Previous research supports that under unbalanced diets, *GAPDH* will have a significant difference with each group [27].

In an earlier report, mice could take up to 750 mg/kg BW of soy peptides from FSBM co-fermented by *Bacillus* spp., *Lactobacillus* spp., and *Saccharomyces* spp., and thus have upregulated *Nrf2* and ARE-related downstream genes expression to reduce the oxidative stress after the strenuous exercise [4]. The results mentioned in Table 7 correspond with those of Figures 2a and 2b. Although the FSBM_B+L_ group did not show the highest CAT activity in the jejunum, the *CAT* was upregulated about 5 times compared to the other groups, which is beneficial to the host in improving the self-antioxidant capacity. The review published by our lab [28] indicated that *Nrf2* is necessary for the host to regulate oxidative stress. After phosphorylation of Nrf2, the activating ARE gene regions synthesize antioxidant-related enzymes (CAT, SOD, etc.) and detoxification-related enzyme (HO-1). If the host is exposed to excessive oxidative stress, it may inhibit the activity of the *Nrf2* pathway and reduced the self-antioxidant capacity.

On the other hand, we also examined the regulation of *PepT1* in the jejunum. PepT1 is widely distributed on the gut epithelium, and mainly transports di- or tri-peptides. The expression of *PepT1* was found to be affected by proton gradient and membrane potential [29]. As the absorbing region, the *PepT1* expression in jejunum is affected by the increase in the content of soy peptides, to further help in improving the transport efficiency. If the un-fermented SBM is not fully digested by pepsin and trypsin, it leads to an increase in the allergen content of SBM including Gly m Bd 30K, Gly m Bd 60K, Gly m Bd 28K, and the metabolic peptides produced by pathogens [30] pass through with the aid of PepT1 to cause innate inflammation. In our result, through the mRNA expression analysis, we observed that allergens and others anti-nutrition factors supporting hindgut fermentation were precluded by fermentation. Thus, PepT1 preferentially transported soy peptides instead of di- or tri-peptides from allergens or pathogen fragments that cause inflammation.

## CONCLUSION

The one-stage FSBM by Bv or two-stage fermented soybean meal by Bv and Lb increased the soy peptide contents and significantly improved free radical scavenging ability. When passing through the jejunum, these FSBMs could regulate the distal intestinal microflora and increase the antioxidant gene expression, enabling the host to respond better to potential oxidative stress. Future studies still needed with more field trials to support the positive correlation between potential FSBM derived peptides in the broilers.

## Figures and Tables

**Table 1:** Enzyme activity of one or two-stage fermented soybean meal products

Items (U/g)	FSBM_L_^1)^	FSBM_B_^1)^	FSBM_B+L_^1)^	SEM	p-value
Protease	ND	4.79	3.98	0.66	0.432
α-galactosidase	ND	8.87	5.25	2.25	0.318
Cellulase	2.27^b^	2.51^b^	2.88^a^	0.09	0.012
Mannanase	ND	6.27	5.99	0.77	0.842
Xylanase	0.93	3.03	2.34	0.42	0.065
β-glucanase	2.95	3.35	3.87	0.43	0.352

Each value represents the mean with standard error of mean (n = 3).

FSBM, fermented soybean meal; SEM, standard error of the means; ND, not detected; SBM, soybean meal.

1) FSBM_L_, commercial control; FSBM_B_, SBM one-stage fermented by Bv; FSBM_B+L_, SBM two-stage fermented by Bv and Lb.

a,b Means within a row with different letters differed significantly (p<0.05).

**Table 2:** Chemical composition of one or two-stage fermented soybean meal products (dried)

Nutrient	SBM	FSBM_L_^1)^	FSBM_B_^1)^	FSBM_B+L_^1)^	SEM	p-value
Composition						
DM (%)	88.4^c^	93.2^a^	88.2^c^	89.9^b^	0.001	<0.001
CP (% DM)	43.0	45.1	50.8	48.1	1.72	0.104
EE (% DM)	1.28	2.77	1.88	1.43	0.38	0.054
Ash (% DM)	7.31	7.40	8.75	7.92	0.40	0.077
NDF (% DM)	17.0^c^	27.2^a^	21.7^b^	28.8^a^	1.11	<0.001
ADF (% DM)	8.44^ab^	6.19^b^	10.84^a^	10.57^a^	1.02	0.018
Calcium (% DM)	0.23^b^	0.26^ab^	0.28^a^	0.28^a^	0.01	0.026
Total phosphorus (% DM)	0.83	0.95	0.91	0.91	0.03	0.086
TCA-soluable protein (mg/g DM)	25.35	25.01	65.86	39.29	3.78	<0.001
A-NSI (% DM)	4.21^d^	7.42^c^	15.17^a^	10.29^b^	0.49	<0.001
Lactic acid bacteria (Log CFU/g DM)	5.54^c^	8.82^a^	8.00^b^	8.97^a^	0.08	<0.001
Anti-nutritive factors						
Raffinose (% DM)	1.29^a^	0.11^b^	0.08^b^	0.03^b^	0.04	<0.001
Stachyose (% DM)	4.15^a^	1.19^b^	ND	ND	0.08	<0.001
Allergen protein (mg/g DM)	505^a^	226^b^	183^b^	198^b^	35.8	0.001
Trypsin inhibitor (mg/g DM)	17.5^a^	9.03^b^	0.43^d^	1.57^c^	0.16	<0.001

Each value represents the mean with standard error of mean (n = 3).

SBM, soybean meal; SEM, standard error of the means; DM, dry matter; CP, crude protein; EE, ether extract; NDF, neutral detergent fiber; ADF, acid detergent fiber; TCA-NSI, Trichloroethanoic acid-nitrogen solubility index; ND, not detected.

1) FSBM_L_, commercial control; FSBM_B_, SBM one-stage fermented by Bv; FSBM_B+L_, SBM two-stage fermented by Bv and Lb.

a–d Means within a row with different letters differed significantly (p<0.05).

**Table 3:** Composition and calculated analysis (% as fed) of the diet for starters broilers (1 to 21 days)

Ingredients	SBM	FSBM_L_^1)^	FSBM_B_^1)^	FSBM_B+L_^1)^
Composition (%)				
Corn, yellow	52.99	53.27	54.18	53.49
Soybean meal (CP, 44%)	34.0	28.0	28.0	28.0
Fermented soybean meal	-	6.0	6.0	6.0
Full fat soybean meal	3.00	2.99	2.00	2.47
Soybean oil	3.16	2.89	2.97	3.20
Fish meal (CP, 65%)	3.00	3.00	3.00	3.00
Monocalcium phosphate	1.32	1.32	1.32	1.32
Calcium carbonate	1.36	1.36	1.36	1.36
NaCl	0.34	0.34	0.34	0.34
DL-Methionine	0.35	0.35	0.35	0.35
L-Lysine HCl	0.20	0.20	0.20	0.20
Choline-Cl	0.08	0.08	0.08	0.08
Vitamin premix^2)^	0.10	0.10	0.10	0.10
Mineral premix^3)^	0.10	0.10	0.10	0.10
Total	100.0	100.0	100.0	100.0
Calculated nutrient levels				
CP (%)	23.0	23.0	23.0	23.0
Crude fat (%)	6.6	6.4	6.3	6.6
ME (kcal/kg)	3,050.0	3,050.0	3,050.0	3,050.0
Calcium (%)	0.96	0.96	0.96	0.96
Total phosphorus (%)	0.70	0.71	0.71	0.71
Non-phytate phosphorus (%)	0.48	0.47	0.46	0.46
Methionine+cysteine (%)	1.08	1.08	1.07	1.07
Chemical analysis value				
DM (%)	10.4	10.6	11.0	10.2
CP (%)	21.6	23.2	23.0	23.0

SBM, soybean meal; CP, crude protein; ME, metabolizable energy; DM, dry matter.

1) FSBM_L_, commercial control; FSBM_B_, SBM one-stage fermented by Bv; FSBM_B+L_, SBM two-stage fermented by Bv and Lb.

2) Vitamins (premix content per kg diet): Vit. A, 15,000 IU; Vit. D_3_, 3,000 IU; Vit. E, 30 mg; Vit. K_3_, 4 mg; thiamine, 3 mg; riboflavin, 8 mg; pyridoxine, 5 mg; Vit. B_12_, 25 μg; Ca-pantothenate, 19 mg; niacin, 50 mg; folic acid, 1.5 mg; and biotin, 60 μg.

3) Minerals (premix content per kg diet): Co (CoCO_3_), 0.255 mg; Cu (CuSO_4_·5H_2_O), 10.8 mg; Fe (FeSO_4_·H_2_O), 90 mg; Mn (MnSO_4_·H_2_O), 90 mg; Zn (ZnO), 68.4 mg; Se (Na_2_SeO_3_), 0.18 mg.

**Table 3:** Composition and calculated analysis (% as fed) of the diet for finisher broilers (22 to 35 days)

Ingredients	SBM	FSBM_L_^1)^	FSBM_B_^1)^	FSBM_B+L_^1)^
Composition (%)				
Corn, yellow	57.15	57.39	58.45	57.61
Soybean meal (CP, 44%)	28.0	22.0	22.0	22.0
Fermented soybean meal	-	6.0	6.0	6.0
Full fat soybean meal	4.15	4.18	3.04	3.65
Soybean oil	4.13	3.86	3.93	4.17
Fish meal (CP, 65%)	3.00	3.00	3.00	3.00
Monocalcium phosphate	1.25	1.25	1.25	1.25
Calcium carbonate	1.28	1.28	1.28	1.28
NaCl	0.34	0.34	0.34	0.34
DL-Methionine	0.27	0.27	0.27	0.27
L-Lysine HCl	0.16	0.16	0.16	0.16
Choline-Cl	0.08	0.08	0.08	0.08
Vitamin premix^2)^	0.10	0.10	0.10	0.10
Mineral premix^3)^	0.10	0.10	0.10	0.10
Total	100.0	100.0	100.0	100.0
Calculated nutrient levels				
Crude protein (%)	21.0	21.0	21.0	21.0
Crude fat (%)	7.8	7.7	7.6	7.8
ME (kcal/kg)	3,175.0	3,175.0	3,175.0	3,175.0
Calcium (%)	0.90	0.91	0.91	0.91
Total phosphorus (%)	0.66	0.68	0.67	0.67
Non-phytate phosphorus (%)	0.46	0.44	0.44	0.44
Methionine+Cysteine (%)	0.95	0.95	0.94	0.95
Chemical analysis value				
DM (%)	10.7	9.4	10.9	10.2
CP (%)	20.5	21.1	21.6	21.6

SBM, soybean meal; CP, crude protein; DM, dry matter.

1) FSBM_L_, commercial control; FSBM_B_, SBM one-stage fermented by Bv; FSBM_B+L_, SBM two-stage fermented by Bv and Lb.

2) Vitamins (premix content per kg diet): Vit. A, 15,000 IU; Vit. D_3_, 3,000 IU; Vit. E, 30 mg; Vit. K_3_, 4 mg; thiamine, 3 mg; riboflavin, 8 mg; pyridoxine, 5 mg; Vit. B_12_, 25 μg; Ca-pantothenate, 19 mg; niacin, 50 mg; folic acid, 1.5 mg; and biotin, 60 μg.

3) Minerals (premix content per kg diet): Co (CoCO3), 0.255 mg; Cu (CuSO_4_·5H_2_O), 10.8 mg; Fe (FeSO_4_·H_2_O), 90 mg; Mn (MnSO_4_·H_2_O), 90 mg; Zn (ZnO), 68.4 mg; Se (Na_2_SeO_3_), 0.18 mg.

**Table 4:** Effect of partial replacement of diet with fermented soybean meal on growth performance of broilers

Items	Experimental diets^1)^				SEM	p-value

	SBM	FSBM_L_	FSBM_B_	FSBM_B+L_		
1–21 d						
Body weight (g/bird)	927	903	928	970	22.0	0.238
Feed intake (g/bird)	1,156	1,106	1,153	1,186	33.9	0.445
Weight gain (g/bird)	882	859	883	926	21.9	0.228
FCR^2)^	1.32	1.29	1.31	1.28	0.04	0.878
22–35 d						
Body weight (g/bird)	2,171	2,177	2,136	2,252	116	0.911
Feed intake (g/bird)	2,052	2,166	2,172	2,099	138	0.913
Weight gain (g/bird)	1,245	1,275	1,209	1,282	109	0.962
FCR^2)^	1.65	1.70	1.80	1.64	0.07	0.285
1–35 d						
Feed intake (g/bird)	3,208	3,272	3,325	3,285	144	0.950
Weight gain (g/bird)	2,126	2,134	2,091	2,208	116	0.907
FCR^2)^	1.51	1.53	1.59	1.49	0.04	0.217
Survival rate (%)	88.8	95	93.8	98.8	5.22	0.611
Performance efficiency factor^3)^	366	385	414	428	33.3	0.588

Each value represents the mean with standard error of mean (n = 4).

SBM, soybean meal; SEM, standard error of the means; FCR, feed conversion rate.

1) FSBM_L_, commercial control; FSBM_B_, SBM one-stage fermented by Bv; FSBM_B+L_, SBM two-stage fermented by Bv and Lb.

2) Feed conversion = feed intake/weight gain.

3) Performance efficiency factor = [(BW×survival rate (%))/(FCR×days of age)]×100

**Table 5:** Effect of partial replacement of diet with fermented soybean meal on blood biochemistry of 35 d-old broilers

Items	Experimental diet^1)^				SEM	p-value

	SBM	FSBM_L_	FSBM_B_	FSBM_B+L_		
GLU (mg/dL)	207^b^	251^a^	242^a^	237^a^	7.48	0.002
BUN (mg/dL)	1.25	1.00	1.13	1.00	0.11	0.305
UA (mg/dL)	3.74	3.75	3.53	3.26	0.40	0.817
SGOT (U/L)	291	322	271	290	33.7	0.752
SGPT (U/L)	13.8	16.6	14.5	15.4	1.58	0.610
T-P (g/dL)	2.85	2.75	2.66	2.69	0.06	0.108
ALB (g/dL)	1.35	1.29	1.26	1.29	0.03	0.335
GLO (g/dL)	1.5	1.45	1.40	1.40	0.03	0.112
AlkP (IU/L)	1,720	1,844	2,055	1,779	134	0.314
CHOL (mg/dL)	117	116	109	117	5.64	0.755
HDL-C (mg/dL)	79.4	83.6	77.8	84.1	3.31	0.467
LDL-C (mg/dL)	28.0	25.1	24.5	23.4	1.90	0.398

Each value represents the mean with standard error of mean (n = 8).

SBM, soybean meal; SEM, standard error of the means; GLU, glucose; BUN, blood urea nitrogen; UA, urea; SGOT, serum glutamic-oxalocetic transaminase; SGPT, serum glutamic-pyruvic transaminase; T-P, total protein; ALB, albumin; GLO, hgobulin; AlkP, alkaline phosphatase; CHOL, cholesterol; HDL-C, high density lipoprotein-cholesterol; LDL-C, low density lipoprotein cholesterol.

1) FSBM_L_, commercial control; FSBM_B_, SBM one-stage fermented by Bv; FSBM_B+L_, SBM two-stage fermented by Bv and Lb.

a,b Means within a row with different letters differed significantly (p<0.05).

**Table 6:** Effect of partial replacement of diet with fermented soybean meal on intestinal microflora concentration of 35 d-old broilers

Microbial parameter (log CFU/g)	Experimental diet^1)^				SEM	p-value

	SBM	FSBM_L_	FSBM_B_	FSBM_B+L_		
Lactic acid bacteria						
Ileum	8.00	8.52	8.39	8.00	0.26	0.419
Cecum	8.10^ab^	7.85^b^	8.69^a^	8.80^a^	0.25	0.031
Coliform						
Ileum	7.00	7.62	7.91	7.35	0.35	0.471
Cecum	7.95^a^	8.2^a^	7.83^a^	7.22^b^	0.19	0.019
L/C ratio						
Ileum	1.19	1.15	1.06	1.08	0.07	0.622
Cecum	1.04^b^	0.95^b^	1.11^ab^	1.22^a^	0.05	0.025

Each value represents the mean with standard error of mean (n = 6).

CFU, colony-forming unit; SBM, Soybean meal; SEM, standard error of the means.

1) FSBM_L_, commercial control; FSBM_B_, SBM one-stage fermented by Bv; FSBM_B+L_, SBM two-stage fermented by Bv and Lb.

a,b Means within a row with different letters differed significantly (p<0.05).

**Table 7:** Effect of partial replacement of diet with fermented soybean meal on serum, jejunum’ s antioxidant activity of 35d-old broilers

Items	Experimental diet^1)^				SEM	p-value

	SBM	FSBM_L_	FSBM_B_	FSBM_B+L_		
Jejunum						
SOD (U/mg protein)	32.4^b^	48.4^ab^	44.8^ab^	63.1^a^	5.61	0.030
CAT (nmol/min/mg protein)	598^c^	841^b^	1,162^a^	817^b^	48.9	<0.001
GSH-Px (nmol/min/mg protein)	7.26	5.61	6.73	6.64	1.23	0.781
MDA (μM)	6.94	11.9	8.61	8.60	1.11	0.067
Serum						
SOD (U/mL)	46.9	36.2	52.1	40.1	7.64	0.439
CAT (nmol/min/mL)	372^b^	346^b^	693^a^	697^a^	39.6	<0.001
GSH-Px (nmol/min/mL)	39.9	45.8	34.4	43.3	9.27	0.803
MDA (μM)	5.84	13.9	11.9	14.2	2.22	0.092

Each value represents the mean with standard error of mean (n = 5).

SBM, soybean meal; SEM, standard error of the means; SOD, superoxide dismutase; CAT, catalase; GSH-Px, glutathione peroxidase; MDA, malondialdehyde.

1) FSBM_L_, commercial control; FSBM_B_, SBM one-stage fermented by Bv; FSBM_B+L_, SBM two-stage fermented by Bv and Lb.

a–c Means within a row with different letters differed significantly (p<0.05).

## References

[1] He L, Han M, Qiao S (2015). Soybean antigen proteins and their intestinal sensitization activities. Curr Protein Pept Sci.

[2] Sanjukta S, Rai AK (2016). Production of bioactive peptides during soybean fermentation and their potential health benefits. Trends Food Sci Technol.

[3] Ashaolu TJ (2020). Health applications of soy protein hydrolysates. Int J Pept Res Ther.

[4] Cui J, Xia P, Zhang L, Hu Y, Xie Q, Xiang H (2020). A novel fermented soybean, inoculated with selected Bacillus, Lactobacillus and Hansenula strains, showed strong antioxidant and anti-fatigue potential activity. Food Chem.

[5] Yang J, Wu X, Chen H, Sun-waterhouse D, Zhong H, Cui C (2019). A value-added approach to improve the nutritional quality of soybean meal by-product: Enhancing its antioxidant activity through fermentation by Bacillus amyloliquefaciens SWJS22. Food Chem.

[6] Chachaj R, Sembratowicz I, Krauze M, Ognik K (2019). The effect of partial replacement of soybean meal with fermented soybean meal on chicken performance and immune status. J Anim Feed Sci.

[7] Wu P, Golly MK, Guo Y (2020). Effect of partial replacement of soybean meal with high-temperature fermented soybean meal in antibiotic-growth-promoter-free diets on growth performance, organ weights, serum indexes, intestinal flora and histomorphology of broiler chickens. Anim Feed Sci Technol.

[8] Toe CJ, Foo HL, Loh TC, Mohamad R, Rahim RA, Idrus Z (2019). Extracellular proteolytic activity and amino acid production by lactic acid bacteria isolated from Malaysian foods. Int J Mol Sci.

[9] Hati S, Vij S, Mandal S, Malik RK, Kumari V, Khetra Y (2014). α-Galactosidase activity and oligosaccharides utilization by lactobacilli during fermentation of soy milk. J Food Process Preserv.

[10] Miller GL (1959). Use of dinitrosalicylic acid reagent for determination of reducing sugar. Anal Chem.

[11] Latimer GW (2012). AOAC International Official methods of analysis of AOAC International.

[12] Xie J, Du M, Shen M, Wu T, Lin L (2019). Physico-chemical properties, antioxidant activities and angiotensin-I converting enzyme inhibitory of protein hydrolysates from Mung bean (Vigna radiate). Food Chem.

[13] Yin L, Tai HM, Lee HH, Jiang ST (2014). Proteolysis and Lactobacillus fermentation effects on the isoflavones biotransformation and removal of anti-nutritional factors of soy bean. J Mar Sci Technol.

[14] Handa CL, de Lima FS, Guelfi MFG, Georgetti SR, Ida EI (2016). Multi-response optimisation of the extraction solvent system for phenolics and antioxidant activities from fermented soy flour using a simplex-centroid design. Food Chem.

[15] Oyaizu M (1986). Studies on products of browning reaction: Antioxidative activities of products of browning reaction prepared from glucosamine. Jpn J Nutr.

[16] Gyamfi MA, Yonamine M, Aniya Y (1999). Free-radical scavenging action of medicinal herbs from Ghana Thonningia sanguinea on experimentally-induced liver injuries. Gen Pharmacol.

[17] Yuris A, Siow L (2014). Comparative study of the antioxidant properties of three pineapple (Ananas comosus L.) Varieties. J Food Stud.

[18] NRC (1994). Nutrient requirements of poultry.

[19] Murugan M, Ragavan A (2017). Broiler performance efficiency factor (BPEF) in commercial broiler production facilities with special reference to climate. Indian Vet J.

[20] Liu Z, Guan X, Zhong X, Zhou X, Yang F (2021). Bacillus velezensis DP-2 isolated from douchi and its application in soybean meal fermentation. J Sci Food Agric 2020.

[21] Zhu J, Gao M, Zhang R (2017). Effects of soybean meal fermented by L. plantarum, B. subtilis and S. cerevisieae on growth, immune function and intestinal morphology in weaned piglets. Microb Cell Fact.

[22] Ketnawa S, Ogawa Y (2018). Comparative study on protein digestibility, protein patterns, antioxidant activities of raw, cooked and fermented soybeans. J Food Sci Agric Technol.

[23] Mukherjee R, Chakraborty R, Dutta A (2016). Role of fermentation in improving nutritional quality of soybean meal-a review. Asian-Australas J Anim Sci.

[24] Alikwe PCN, Faremi AY, Egwaikhide PA (2010). Biochemical evaluation of serum metabolites, enzymes and haematological indices of broiler-chicks fed with varying levels of rumen epithelial scraps in place of fish meal proteins. Res J Poult Sci.

[25] Apajalahti J, Vienola K (2016). Interaction between chicken intestinal microbiota and protein digestion. Anim Feed Sci Technol.

[26] Soumeh EA, Mohebodini H, Toghyani M, Shabani A, Ashayerizadeh A, Jazi V (2019). Synergistic effects of fermented soybean meal and mannan-oligosaccharide on growth performance, digestive functions, and hepatic gene expression in broiler chickens. Poult Sci.

[27] Guo S, Zhang Y, Cheng Q (2020). Partial substitution of fermented soybean meal for soybean meal influences the carcass traits and meat. Animals.

[28] Lee MT, Lin WC, Lee TT (2019). Potential crosstalk of oxidative stress and immune response in poultry through phytochemicals-a review. Asian-Australas J Anim Sci.

[29] Karaś M (2019). Influence of physiological and chemical factors on the absorption of bioactive peptides. Int J Food Sci Technol.

[30] Ayyadurai S, Charania MA, Xiao B, Viennois E, Merlin D (2013). PepT1 expressed in immune cells has an important role in promoting the immune response during experimentally induced colitis. Lab Invest.

